# Disaster governance, energy insecurity, and public health in rural Puerto Rico: how communities resist political abandonment

**DOI:** 10.3389/fpubh.2025.1595830

**Published:** 2025-07-11

**Authors:** Sergio Rivera-Rodríguez, Adrian Santiago-Santiago, Sheilla R-Madera, Mark Padilla, Nelson Varas-Díaz, Kariela Rivera-Bustelo, Claudia Mercado-Rios, John Vertovec, Arturo Massol-Deyá, Jeffrey Ramos, Genevieve Reid, Rebecca Rodríguez-Banch, Kevin Grove, Emil Varas-Rodríguez

**Affiliations:** ^1^CUNY School of Public Health, New York, NY, United States; ^2^Ponce Health Sciences University, Ponce, PR, United States; ^3^Florida International University, Miami, FL, United States; ^4^University of Puerto Rico Mayagüez, Mayagüez, PR, United States; ^5^University of Rochester Medical Center, Rochester, NY, United States; ^6^Mayo Clinic, Rochester, MN, United States

**Keywords:** disasters, political abandonment, energy insecurity, solar power, rural communities, aging population, community organization

## Abstract

Puerto Rico, an unincorporated US territory, faces systemic governmental neglect that disproportionately affects public health, particularly in the wake of disasters. Systemic patterns of political corruption, and post-disaster mismanagement, rooted in colonial governance, have shaped PR’s longstanding vulnerability reflecting in frequent power outages and energy delays. This study examines political abandonment feelings in Puerto Rico through the lens of energy insecurity and disaster governance, focusing on the rural municipality of Adjuntas. Using Atiles’ framework of Normalization of Exceptionality and (In)Security, we investigate how state inaction, privatization, and bureaucratic inefficiencies perpetuate vulnerability in disaster-prone communities. Using qualitative in depth-interviews with Adjuntas’ residents living with chronic conditions (*n* = 45) and stakeholders (*n* = 15), we explore the impact of political abandonment in energy instability. We employed thematic analysis to identify patterns and themes within the dataset. We focus on three primary themes: feelings of political abandonment, political corruption, and community response. Many participants expressed feeling abandoned by the government, particularly in the wake of energy crises and disaster recovery failures. Our findings reveal that political abandonment is not merely a failure of governance but an intentional strategy that limits recovery efforts and sustains structural inequalities. The privatization of PR’s electrical grid has exacerbated disparities, reinforcing patterns of disaster capitalism. Casa Pueblo, a community organization, demonstrate community-driven resilience, offering a model of decentralized energy solutions that counteract state neglect. These findings contribute to broader discussions on disaster governance, energy justice, and health disparities.

## 1 Introduction

Puerto Rico (PR), an unincorporated US territory, faces systemic governmental neglect that disproportionately affects public health, particularly in the wake of disasters. This neglect is rooted in colonial governance (36), which has shaped PR’s longstanding vulnerability. As a Caribbean archipelago, PR is highly susceptible to hurricanes (e.g., María, 2017; Fiona, 2022), earthquakes, floods and droughts that have severely strained its already fragile health and energy infrastructure (38). Decades of economic decline—worsened by the 2016 debt crisis and austerity measures under the PROMESA law (i.e., downsizing government agencies, awards to outside consultants and lobbyists, cuts in government services) have further eroded these essential systems (1, 37).

Hurricane María exposed this structural collapse, leading to approximately 3,000 deaths (2), not from the storm itself but due to the failure of the healthcare system and prolonged power outages that disrupted hospital operations, access to life-saving treatments, and medical equipment (3). One month after the hurricane, 36% of the population still lacked access to water, affecting nearly 1 million people and further straining medical and mental health services in rural PR (4, 5). The storm also devastated the electric grid, disabling 80% of transmission lines and leaving 100% of the population without energy (4).

These cascading crises— energy insecurity (6), healthcare system collapse (3), and economic instability (7)—have created an ongoing public health emergency. Structural issues such as the mass migration of healthcare professionals (8) and PR’s colonial status (36) further exacerbate PR’s vulnerability, limiting the archipelago’s ability to recover and prepare for future disasters. Unlike independent Caribbean nations, PR’s lack of fiscal autonomy and constrained disaster response capacity reinforces cycles of governmental neglect and abandonment.

Compounding these challenges, a lack of disaster preparedness, a pervasive fiscal deficit of $74 billion, systemic underinvestment, and political corruption have further weakened PR’s infrastructure (4, 9). Privatization of the energy sector—through contracts with companies like LUMA Energy and Genera PR—has failed to stabilize electric services, leading to increasingly frequent and prolonged blackouts and brownouts despite rising energy costs (10). While the local legislature passed the Puerto Rico Energy Public Policy Act (Act 17) in 2019, which aims for 100% renewable energy by 2050, reports indicate that as of 2024, only 12% of PR’s energy comes from renewable sources—far below the 40% goal set for 2025 (11).

Power outages and energy delays are not isolated failures but reflect systemic patterns of post-disaster mismanagement including governmental neglect and political corruption (12). This study builds on a long-standing body of literature that frames disasters as socially and politically constructed events rather than purely natural phenomena. As emphasized by Quarantelli (13) and Hewitt (14), disasters emerge from underlying conditions of vulnerability shaped by structural inequality, colonialism, and uneven power relations (13, 14). Originating from Klein’s foundational work, this study also explores the concept of disaster capitalism referring to the ways in which crises are leveraged to advance privatization, austerity, and neoliberal restructuring often to the detriment of vulnerable populations (15). Recent scholarship has pushed this concept further, arguing that disaster capitalism is not only a reactive (ex-post) strategy but also anticipatory (ex-ante), shaping infrastructure, governance, and policy decisions long before disasters occur (16).

Our analysis builds on this evolving body of work to show how, in the Puerto Rican case, disaster capitalism operates through both historical neglect and opportunistic post-disaster interventions (17–19). While scholars have examined how neoliberal disaster responses exacerbate vulnerabilities in the Caribbean (20) and how privatization deepens inequalities in post-disaster contexts (15), these dynamics remain understudied in PR, where colonial legacies further entrench disaster governance failures. Although governmental inaction in PR has been documented [e.g., (4)], limited research explores how rural communities directly experience and resist this abandonment, particularly in the face of prolonged energy insecurity, which disproportionately impacts healthcare access and the management of chronic conditions among aging populations (21).

This study addresses this gap by examining how political abandonment is not only experienced but also actively contested by affected communities, offering new insights into state withdrawal in disaster contexts. We focus on Adjuntas, a rural, mountainous municipality, which highlights the vulnerabilities of a population where 21% are over 65 years old and 63.4% lives below the poverty level (22). Additionally, 14.9% of Adjuntas’ residents have a health-related disability, making them particularly susceptible to disruptions in medical care during disasters (22). Research underscores that older adults in rural areas face heightened risks during and after disasters due to their complex medical needs and geographical isolation (23, 33). Bureaucratic inefficiencies and the prioritization of younger populations further exacerbate these risks, limiting older adults’ access to essential resources post-disasters (24).

In response to state inaction, *Casa Pueblo*—a self-sustaining non-profit community organization dedicated to protecting natural, cultural, and human resources—has played a crucial role in addressing energy insecurity and its impact on health. By installing solar panels for residents with chronic conditions in Adjuntas, *Casa Pueblo* has facilitated the management of energy-dependent chronic health conditions (e.g., renal disease, diabetes), offering a model of community resilience (25).

This article draws on Atiles’ (34) framework of *Normalization of Exceptionality and (In)Security* in PR, which argues that disasters are not simply natural phenomena but socially produced crises rooted in slow and structural violence driven by racio-colonial governance. Rather than acting as ‘great levelers,’ disasters exacerbate pre-existing inequalities along race, class, and gender lines. Atiles posits that the perpetual state of emergency and misuse of executive orders in PR have facilitated corruption and disaster capitalism, prioritizing private interest over public welfare. Using this framework, this article expands on Atiles’ conceptualization of continuity of crises to examine how governmental neglect in energy infrastructure perpetuates health crises and contributes the reinforcing Puerto Ricans’ sense of political abandonment while simultaneously giving rise to alternative forms of resistance and self-sufficiency.

## 2 Methods

### 2.1 Study design

This study is part of a broader ongoing exploratory sequential mixed-methods project funded by the National Institutes of Health (Grant No. 1R01AG072613-01A1) on energy independence and chronic disease management. For this article, we focus specifically on the qualitative component, drawing on in-depth semi-structured qualitative interviews (ISQIs) with individuals over 50 living with chronic conditions in Adjuntas, PR. Additionally, we incorporate insights from stakeholder interviews with government officials, business owners, professional organizations, and *Casa Pueblo’s* advisory board to contextualize the findings. The study was approved by the Florida International University Institutional Review Board.

### 2.2 Recruitment and data collection

We collaborated with *Casa Pueblo’s* community liaison to recruit participants from a database of individuals who had received solar panels through the organization. The study included 45 individuals over 50 years old living with chronic conditions in Adjuntas. Participants were selected from 13 neighborhoods in Adjuntas, grouping them based on their level of access to solar energy: (1) *Direct access* (DA): participants with solar panels installed in their homes (*n* = 15); (2) *Indirect access* (IDA): participants without solar panels in their homes but living within a one-mile radius of solar-powered sites (*n* = 15), and (3) *No access* (NA): participants without nearby solar installations (*n* = 15). This categorization allowed us to examine how individuals with chronic conditions experience energy insecurity based on their degree of access to renewable energy. To ensure a diverse sample, we used snowball sampling within each group to recruit additional participants. A summary of participants’ demographic data is included in Table 1. We also conducted 15 stakeholders’ interviews with government officials, business owners, professional organizations, and *Casa Pueblo’s* advisory board. These stakeholders were selected for their involvement in the community, expertise in energy issues, and knowledge on chronic disease management. All participants provided informed consent before participating in the study.

**Table 1:** Participant’s demographic characteristics and solar panel accessibility.

Gender	Number	Percentage
Female	32	71
Male	13	29
Age*		
<50	3	7
50–64	16	36
65–79	14	32
80+	11	25
Race*		
White	28	64
Black	1	2
More than one race	11	25
Prefer not to answer	4	9
Education*		
Did not finish HS	9	21
HS graduate	21	49
College graduate	13	30
Marital status*		
Single	4	9
Married	19	43
Widow/Widower	13	30
Live with partner (not legally married)	4	9
Divorced	3	7
Separated	1	2
Source of income*		
Social security	30	70
Full time job	1	2
Part time job	4	9
Retired/Pensioner	3	7
Other	5	12
Monthly income*		
0–499	10	23
500–999	15	34
1,000–1,499	9	20
1,500–1,999	6	14
2,000+	4	9
Solar panel access		
Direct access	15	33
Indirect access	15	33
No access	15	33

*Variable account for only 44 participants due to incomplete demographic information from one participant (IDA-012).

Interviews were conducted either in participants’ homes or in public spaces of their preference. Each interview lasted between 25 and 60 min and was audio-recorded using iPads. Interviewers were divided into pairs and conducted most ISQIs together to optimize data collection. While one person led the interview, the other person monitored audio quality, took notes, and assisted with follow-up questions. Participants received a $50 cash stipend for their participation.

### 2.3 Measures

We developed an ISQI guide, which included 29 open-ended questions addressing the following topics: (1) challenges faced during power outages and the impact on their health, (2) strategies used to manage power outages and energy insecurity, (3) challenges in adapting to energy independence, and (4) community interactions and support systems. The ISQI also included a sociodemographic data questionnaire. To refine the interview guide, we sought feedback from a specialist in aging with vast experience in the Puerto Rican context.

### 2.4 Data analysis

All interviews were transcribed verbatim for analysis. Since the interviews were conducted in Spanish, the authors translated the results into English for publication. The translators are fully bilingual and fluent in English and Spanish. We employed thematic analysis (35) to identify patterns and themes within the dataset. The qualitative analysis followed a multi-step *in vivo* process: (1) A focused codebook was developed by categorizing themes that emerged from an initial review of preliminary sample transcriptions, (2) Six independent coders worked in pairs to apply the codes to all transcripts. To enhance inter-coder reliability, discrepancies were discussed and resolved through peer debriefing, and a final agreement on coding categories was reached. Final coding agreements were integrated into NVivo qualitative data analysis software, and (3) each coder produced a one-page analytic summary per transcript, highlighting key insights on the final codebook. The research team held biweekly meetings to discuss findings from the analytic summaries and identify salient themes.

## 3 Results

### 3.1 Feelings of political abandonment

The results reveal pervasive feelings of political abandonment among both community members and stakeholders. Participants consistently described government inaction as systemic neglect, reinforcing their perception that the state has withdrawn from its responsibilities toward rural and poor communities. This neglect is particularly evident in PR’s rural regions, where disparities in access to essential services—including healthcare, electricity, and disaster relief—leave entire communities vulnerable. Rural residents consistently reported feeling forgotten by the state, especially in the aftermath of disasters, when resources take longer to arrive, if they arrive at all.

#### 3.1.1 Abandonment of rural and poor communities

Participants reported a clear disparity in resource allocation between urban and rural areas, further exacerbating inequalities. Carmen, a 63-year-old woman living with diabetes, hypertension, and asthma, used her solar panels to support her community by refrigerating medications and sharing energy with others. She expressed frustration with government priorities:

*“The government cares more about those in the city than those in the countryside.” (DA-011, 63, F)*

Other participants expanded on this theme, describing how rural communities receive essential services much later than urban centers after disasters. A 74-year-old participant from the indirect access (IDA) group compared her neighborhood experience to that of wealthier areas:

*“The rural towns are always kind of the last ones. It’s like I was saying, you see that they just turned the lights back on in Condado [a wealthy area of the capital, San Juan] because that’s where the wealthy people are, but we, we went almost 6, nearly 7 months without power in Guilarte [rural town] because it’s a small town where, the people are poor, so, we don’t generate any money. So, we’re the forgotten town, that’s the reality.” (IDA-011, 74, F)*

Poverty was a key concern among participants, many of whom felt that the government ignored poor communities. Adjuntas has one of the highest poverty rates in PR, with over 50% of its population living below the poverty line (26). The average yearly salary in the municipality is $17,659 USD, barely enough to support a basic standard of living (26). Extreme poverty means that some individuals lack basic necessities such as food and safe drinking water, increasing their stress and sense of despair. Several participants linked these conditions of deprivation to deteriorating mental health, noting that many community members experience depression and hopelessness due to persistent neglect. A political official from Adjuntas described how poverty remains hidden in PR, narrating a striking example of extreme neglect:

*“I can tell you that I’ve seen houses with dirt floors. At this point in time. Recently, a lady passed away, and we were all curious, everyone in town was curious, but no one knew what was happening. The lady died, the police called me, and I found it odd. When I went there, I found out that those people had been living without water or electricity since 2011.” (STKH-011, government official)*

For many, this chronic state of abandonment has consequences beyond material deprivation. Ramón, a 63-year-old man living with hypertension, linked rural poverty, political neglect, and mental health, explaining how suicide rates increased because of abandonment:

*“Because there is extreme poverty here in Puerto Rico, you know. But why? Because the government doesn’t get involved where it needs to. You know, they need to get out of “la losa” [referring to wealthy regions], as we say, leave San Juan [PR’s capital], and come to the rural areas, where you can see where the real needs are. There are a lot of sick people. Many people, well, what do they do? They take their own lives because they don’t have help.” (DA-006, 63, M)*

Government officials, in contrast, attributed the situation to a lack of personnel and resources. An Adjuntas government official acknowledged the residents’ abandonment and expressed a desire to improve conditions but pointed out severe understaffing and poor coordination across federal, state, and municipal levels, which hinders intervention efforts. These inefficiencies are particularly evident in the energy sector, where delays in restoring power have left residents in prolonged blackouts, worsening their daily struggles:

*“I’m addressing families in need, but the demand we have for that service is overwhelming, we can’t keep up, we can’t keep up.” (STKH-011, government official)*

#### 3.1.2 Abandonment of projects to improve the energy system

Participants felt trapped in deteriorating conditions as energy prices continued to rise despite poor service quality. The combination of unreliable electricity and escalating costs was particularly concerning for those managing chronic conditions, as power outages disrupted the use of essential medical equipment and refrigeration of medications. A businessman from Adjuntas described the financial strain caused by rising energy prices:

*“Right now, the government [via subcontracting private companies] is just charging, charging, and charging. I know the increases are necessary, but they are kind of too extreme, and most people have low incomes, so it's tough.” (STKH-005, business owner)*

Frustration over high costs and unreliable services led many participants to question why the government had not prioritized solar energy, despite its potential to provide long-term energy security.

*“They’re not prioritizing the solar panels, recycling, those kinds of things. The government isn’t doing what it should. Money is going elsewhere.” (DA-007, 77, F)*

Some suggest that solar energy remains a low priority because the government is overwhelmed by other crises:

*“In my opinion, yes, they should help with solar energy, but with so many unresolved issues—just look at how the Department of Education is in complete disarray—they push the solar energy issue to the side and don’t give it any attention at all.” (DA-009, 82, M)*

Even policymakers agreed that energy projects were being neglected. A state legislator and a municipal legislator confirmed that no active plans exist to advance solar energy:

*“So, the people who need medical equipment, etcetera, I don’t understand why the government hasn’t made a plan that says: let’s set up microgrids to provide and facilitate this, for example, at Centro Médico, primary hospitals, or … (interviewee: yes, critical places) yes, critical places. That hasn’t even been talked about.” (STKH-009, government official)*

*“At the moment, I can tell you that no project related to solar energy has reached the legislature, but yes, the public has sent letters to the legislature expressing concern about that particular issue.” (STKH-012, government official)*

#### 3.1.3 The government benefits from abandonment

Participants overwhelmingly expressed distrust in government officials, arguing that political elites not only neglect energy projects but actively obstruct them to protect corporate interests. Many viewed the government’s inaction as a deliberate strategy to maintain economic dependence and prevent communities from achieving energy self-sufficiency:

*“The problem with the government is that they always want to profit from you. I don’t know if you’ve heard the rumors, but as people are starting to think about solar panels and solar energy, they wanted to charge a tax on the sun, which I find ridiculous because that’s natural, and we shouldn’t be charged for it.” (STKH-004, business owner)*

Beyond government inaction, private corporations also profit from disasters through public contracts. During a conversation with a state legislator, he admitted that even federal public entities like FEMA recognize that private companies exploit disasters for financial gain. Participants saw this dynamic as part of a larger pattern in which the Puerto Rican government prioritizes the interests of politically connected corporations over the needs of its population, especially in the energy sector:

*“I met here with some FEMA officials, and while sitting here, the FEMA official admitted to me that a disaster economy exists, where every time there’s a disaster, all these companies come to make money.” (STK-009, government official)*

This pattern is part of a broader phenomenon in which crises are leveraged to justify privatization and corporate profit—often referred to as ‘disaster capitalism’ (20). The privatization of PR’s energy system, specifically the transfer of operations to LUMA Energy, stands as one of the most significant examples of this model. Participants expressed frustration that, rather than strengthening the public sector to respond to future disasters, the government consistently turns to private entities, further deepening public distrust and reinforcing cycles of abandonment.

Another emerging theme in the interviews was how the government itself acts as a barrier to effective recovery efforts. Participants described excessive bureaucratic inefficiencies that delay aid distribution, especially in rural areas. A municipal government official from Adjuntas highlighted how federal, state, and municipal agencies lack coordination, further slowing emergency response:

*“The other part is the intermediaries. For example, in the cases with FEMA. I have three in the middle. So, you know, it’s not just the regulations we have as a municipality, but I also have to go through the [Central Office of Recovery] and then through the filter of FEMA. Do you know how long that takes?” (STK-011, government official)*

Participants also noted that community organizations provide faster and more effective aid than government agencies, reinforcing a widespread sense of institutional failure. The same municipal official acknowledged this discrepancy:

*“Yes, in distribution, right now, they are announcing where the middle class will benefit, but the problem is that when it's through the Department of Housing, things don’t move as quickly, perhaps because of government bureaucracy. It’s inevitable, but when it's through institutions, in this case in Adjuntas like Casa Pueblo, the aid is much, much faster.” (STK-011, government official)*

This section illustrates the far-reaching implications of political abandonment for rural and low-income communities. Individuals with chronic diseases, already vulnerable due to a deteriorating energy infrastructure, face additional hardships as the government fails to implement necessary policy changes. Moreover, bureaucratic inefficiencies and corporate profit-seeking behaviors contribute to sustained collapse of PR’s energy system. The next section explores how political abandonment intersects with political corruption, further deepening the crisis and shaping the lived experiences of Puerto Ricans.

### 3.2 Political corruption

#### 3.2.1 Feelings toward political corruption

Concerns about political corruption were prevalent among participants, who described it as a central issue affecting governance and public trust. Many community members in Adjuntas expressed frustration with the entrenched corruption, believing that it directly impacts infrastructure projects and resource allocation. Their concerns are supported by official data, which reports 2,574 registered corruption cases in PR’s Department of Justice registry (27).

This high rate of corruption further deepens public distrust, particularly regarding government-led community projects. Participants expressed skepticism toward any government initiatives, fearing that corruption would undermine their execution. When asked how the government could contribute to solar energy projects, one participant responded:

*“Oh! Girl, with all the waste coming from the government. With everything the government is squandering, with all the stealing that's going on… (silence) Even our pensions… that really gets to me. (silence) You know, the government is stealing left and right.” (IDA-006, 64, F)*

Corruption has become a normalized aspect of daily life for people in PR. A recent survey conducted by Ipsos Global ranked corruption as the top concern among Puerto Ricans, surpassing issues such as education, crime, and inflation (28). This sentiment was echoed by some participants, which reflected their deep frustration with how corruption has become an expected part of government:

*“Yes, that’s a word [corruption] that comes up every day. It’s a word that’s used daily, we see it. They imprison this one, imprison that one. They remove another, and it’s all because of corruption.” (IDA-012, M)*

Participants were particularly concerned with the misuse of federal disaster relief funds. PR has received billions of dollars in aid for infrastructure recovery, yet many community members believe that these funds never reach the intended recipients:

“The most serious problem we have in Puerto Rico is that the money always arrives. We know it came, but it never reaches its destination.” (IDA-012, M)

*“Well, with all the millions they are wasting, I think they could provide energy to everyone. Because the amount they waste and steal is brutal—the millions that are being thrown away and squandered.” (NA-004, 68, M)*

The persistence of corruption within energy infrastructure projects, particularly in the post-hurricane reconstruction phase, has led many Puerto Ricans to believe that solar energy projects are deliberately neglected to sustain dependence on private contracts.

#### 3.2.2 Nepotism and private contracts

During the interviews, many participants emphasized how economic opportunities are often distributed based on political loyalty rather than merit. One participant referenced a common Puerto Rican saying that captures this dynamic:

*“He who has no godfather does not get baptized.” (NA-014, 85, M)*

This phrase reflects a widespread belief that political and economic opportunities in PR depend on personal and political connections rather than qualifications or public need. Participants frequently cited instances where government contracts were awarded to politically connected companies at inflated costs:

“Sometimes there are millions allocated to many other areas that really end up in places they shouldn’t. You know, they end up with friends and in contracts with friends who sometimes build things without a real purpose, and it’s more about aesthetics. Sometimes they spend more dollars and millions on that than on something that could really work and give peace and tranquility to many people.” (DA-001, 71, F)

*“They are giving multimillion-dollar projects to companies when maybe things could be done for much less. That’s where the money goes, in those bridge projects and things. Sure, they need to be done, but maybe not so expensive. And that’s how the money disappears, they're stealing the money for other things, and what they should be prioritizing, they aren't doing.” (DA-007, 77, F)*

The privatization of PR’s energy system was carried out with minimal transparency, particularly during a period when the population was focused on disaster recovery (19). Participants expressed deep skepticism toward LUMA Energy, the private company contracted to manage the archipelago’s electrical grid, describing its operations as an extension of political favoritism:

*“No, what the government wants is to collect and try to give money so that LUMA fixes whatever they tell them. Because how long has it been already? And nothing improves, everything gets worse. If you could at least see an intention to improve, but no. I haven't seen any intention to improve here; everything is worse… It’s because of friends. Unfortunately, that’s what this country runs on. Its foundation is that when someone rises, everyone owes something to someone else and has to pay it back somehow, because LUMA fell from the sky. LUMA fell from the sky, and they are the friends of friends working, and we are the ones paying for their luxuries.” (IDA-002, 55, F)*

Beyond infrastructure contracts, nepotism also extended to disaster relief aid. Participants reported that government officials selectively distributed emergency resources based on political affiliations rather than actual need:

*“I saw a government official passing by distributing water and telling us, “don’t give that one anything” or running by to avoid distributing to the people. I saw it because I lived it, I saw people from their [political] party distributing there, already with names and surnames, delivering to the people, but they never came here.” (NA-001, 67, M)*

These findings illustrate how political favoritism reinforces structural inequalities, disproportionately affecting marginalized communities and delaying meaningful recovery efforts.

#### 3.2.3 Federal distrust

The entrenched political corruption and nepotism in PR has eroded trust between the federal and local governments. Participants and stakeholders described a longstanding pattern of mismanagement of federal disaster aid, leading to delays, inefficiencies, and, in some cases, the return of unspent funds to the federal government. For example, in 2022 the Puerto Rican government returned $85 million in federal funds that had been allocated to assist residents with utility payments following the COVID-19 pandemic (29). Participants and government officials alike expressed frustration at the bureaucratic inefficiencies that prevent timely distribution of federal aid:

*“The federal government has a policy that I totally agree with, which is, if I assign you funds for a certain date, it’s for you to use them, not to just keep them stored away. Unfortunately, in Puerto Rico, everything goes through intermediaries, and there’s no planning to have things executed by the assigned date.” (STK-011, government official)*

As a result of ongoing financial mismanagement, the federal government has begun shifting disaster response funding directly to community organizations rather than local government agencies. A participant described this shift:

*“The Department of Housing has those funds. But there are other funds that Biden allocated, 1 billion dollars, also for that same purpose, which are held by the Federal Energy Department. They have not yet determined how to use them. They have already stated that they will not give them to the government of Puerto Rico. They want that money to go directly to community projects. So, they are setting up the framework to move those 1 billion dollars through community organizations for that same purpose.” (STKH-009, government official)*

These findings indicate that political corruption, nepotism, and federal distrust have severely undermined public confidence in government institutions, strengthening the sense of abandonment. These structural failures directly impact the development of solar energy projects, as inefficiencies and mismanagement intensify energy insecurity.

### 3.3 Community response

#### 3.3.1 Coming together outside of political spheres

Many participants emphasized the importance of community-driven action over reliance on political institutions. They expressed confidence in the people of Adjuntas to implement energy solutions independently and believed that meaningful change is more likely to emerge from grassroots initiatives rather than government intervention. Several participants advocated for community ownership of energy projects, emphasizing collaborative efforts with local organizations instead of waiting for government action:

*“We would like for many people to take the initiative to keep pushing this project [solar energization] forward. If it’s possible to completely free ourselves from the abuse. Electricity is a necessity, not a luxury. Now we depend on it for everything, because even if you want a little cold water, you need a refrigerator, which is another expense (laughs). But we need to use solar panels.” (DA-004, 60, F)*

Participants highlighted the success of *Casa Pueblo’s* solar energy initiatives as a key example of how grassroots efforts can drive significant change. They stressed the importance of expanding these initiatives to ensure broader community access to solar energy:

*“With many people coming together, like they’ve done here at Casa Pueblo, they’ve partnered with local businesses, and there are already some places in town that have solar power. The same should be done with people so that the service can reach them.” (DA-013, 65, F)*

When asked how the community could achieve greater access to solar energy, one participant responded:

*“I understand that if Adjuntas could unite, without any politics involved, because they quickly bring in politics, even for that [solar energy projects]. We can come together and try to find alternatives.” (IDA-006, 64, F)*

*Casa Pueblo’s* initiative to install solar panels in local homes has fostered optimism, demonstrating that alternative energy solutions are feasible and effective. Many participants noted that having solar panels not only improved their quality of life but also enhanced their sense of security, health, and overall well-being. One participant reflected on the contrast between governmental inaction and *Casa Pueblo’s* impact:

*“The mayor never dealt with the situation to handle the solar panels. And thanks to Casa Pueblo, this community has all the solar panels that were never seen before.” (DA-014, 62, F)*

This recognition extended to government officials, who acknowledged *Casa Pueblo’s* role in providing sustainable energy solutions where government efforts had failed:

*“In the case of the aid given to people, right, with chronic conditions, the work being done by Casa Pueblo is entirely humanitarian. At least, that's how I see it from my perspective…I have witnessed many people who have benefited from the way Casa Pueblo has provided the fireflies [houses with solar panels], or whatever they call them, in benefit of people in great need.” (STK-011, government official)*

Beyond energy independence, *Casa Pueblo* has also addressed healthcare access gaps. During Hurricane Fiona, the organization collaborated with health professionals to ensure residents received medical services, filling yet another critical void left by the government. One participant described *Casa Pueblo’s* intervention during the hurricane:

*“It has always been forgotten because neither now nor during Fiona did, they [the government] show up there, not even to bring water. Casa Pueblo came in with doctors, they came here recently. They brought us water; they even brought us medicine and prescribed it for my wife and me.” (DA-006, 63, M)*

The community response in Adjuntas highlights the power of grassroots initiatives and collective action in addressing political abandonment. Residents expressed a strong desire to unite outside political spheres, reflecting a growing awareness of their capacity to create sustainable solutions through self-organized efforts. *Casa Pueblo’s* success demonstrates that resilience and progress can emerge from within communities, offering a model of empowerment and sustainable development amid persistent governmental failures.

## 4 Discussion

Our findings indicate that political abandonment in PR is not merely a manifestation of governmental inefficiency but a structural issue that perpetuates vulnerability in rural communities within disaster contexts. By analyzing the energy crisis and its repercussions on public health, it becomes evident that state inaction follows a governance model based on the normalization of prolonged crises. These findings align with Atiles’ (34) argument that disasters in PR are not isolated events but rather the result of long-term structural violence, rooted in a colonial political economy that institutionalizes insecurity as a mode of governance. Poverty is hidden by political entities, taking advantage of the population dilution in rural areas relative to the urban population density.

Using this framework, our results suggest that political abandonment is not just a failure but an active policy choice that limits recovery efforts and constrains PR’s capacity to respond to future crises. The participants’ narratives highlight how the state actively withdraws from core responsibilities, forcing communities to navigate persistent insecurities independently (34). This is particularly evident in the case of energy insecurity, where the privatization of PR’s electrical grid has deepened social inequalities rather than providing stability (12). The transition to LUMA Energy exemplifies a broader pattern of disaster capitalism (19), where crises become opportunities for private entities to profit while public institutions remain deliberately ineffective.

Furthermore, our study underscores the role of bureaucratic inefficiencies in exacerbating political abandonment. Participants consistently described the Puerto Rican government as not just ineffective but actively obstructive in disaster recovery efforts, particularly in the distribution of federal aid. The mismanagement of energy recovery incentives has deepened mistrust between the federal and local governments, which sustains colonial patterns of external oversights and dependence. These bureaucratic failures contribute to the normalization of disaster-driven governance, where marginalized communities are left to manage crises autonomously.

Our findings also align with existing literature on disaster response in PR, particularly regarding the collapse of the health system following Hurricane María (3). Studies have documented how bureaucratic red tape slowed medical aid distribution, leaving individuals with chronic conditions without necessary care (30). Similarly, our research suggests that energy instability—exacerbated by privatization—functions as a structural determinant of health, disproportionately impacting older adults and those managing chronic conditions.

Moreover, participants described stark inequalities in resource allocation, particularly between urban and rural areas. The delayed restoration of electricity in rural municipalities like Adjuntas, compared to wealthier urban centers, reflects a pattern of economic and racialized neglect (31). As Atiles (34) posits, disasters exacerbate pre-existing social hierarchies rather than acting as “great levelers.” This dynamic is particularly evident in PR’s colonial status, where a lack of fiscal autonomy and constrained disaster response capacity leads to cycles of governmental neglect and abandonment.

Despite these systemic failures, our findings emphasize the power of grassroots resilience. *Casa Pueblo’s* community-led solar energy projects challenge the state’s neglect, offering a counterpoint to disaster capitalism. Unlike the privatized energy grid, which has failed to provide stable electricity, *Casa Pueblo’s* model demonstrates how energy independence can be reclaimed at a community level. By promoting local energy sovereignty, *Casa Pueblo* not only mitigates the effects of systemic neglect but also presents a form of decolonial resistance that reclaims power over essential resources. These findings are congruent with research highlighting how Puerto Rican grassroots initiatives have historically resisted colonial governance and disaster mismanagement (3).

Ultimately, this study contributes to the growing field of public health disaster governance by illustrating how political abandonment operates as a determinant of health, intersecting with energy justice and disaster capitalism to exacerbate social inequalities. Our findings emphasize the urgent need for policies that integrate energy justice and disaster resilience into public health planning. Future research should further explore the long-term implications of decentralized energy systems and the potential for community-led initiatives to improve health outcomes and reduce vulnerabilities in disaster-prone regions.

## 5 Conclusion

This study illustrates the profound public health consequences of political abandonment in PR, particularly in rural and economically marginalized communities like Adjuntas. Using Atiles’ (34) framework of *Normalization of Exceptionality and (In)Security*, we found that energy insecurity, healthcare system collapse, and disaster mismanagement are not isolated failures but systemic mechanisms of governance that sustain public health disparities. Our findings highlight how government inaction, privatization, and corruption collectively nurture a disaster economy, disproportionately affecting those most vulnerable, including individuals with chronic conditions.

This study, while offering valuable insights, is subject to certain limitations. First, the data is geographically limited to the municipality of Adjuntas, and while it provides in-depth insights into the local dynamics of political abandonment and community resilience, the findings may not be generalizable to other regions of Puerto Rico. Second, the study captures a particular moment in time and does not account for how these dynamics may evolve in response to shifting political, environmental, or economic conditions. Future research should consider comparative analyses across multiple municipalities to explore regional variations in disaster governance and resistance.

Furthermore, this study contributes to existing literature on disaster capitalism by providing a qualitative, community-based perspective on how Puerto Ricans experience and resist systemic failures. The privatization of PR’s energy grid, coupled with deliberate bureaucratic inefficiencies in energy recovery projects, illustrates how governments and corporations exploit crises for financial gain, which prolongs cycles of insecurity and dependance on privatized services (12, 19). Additionally, this study aligns with the approach of Sandoval et al. (32), which contends that disaster capitalism is not limited to the aftermath of a catastrophe but is also embedded in the conditions that precede it (Sandoval, 2022). This is evident in the Puerto Rican context through long-standing colonial austerity and the systematic underinvestment in critical infrastructure that heightens community vulnerability even before a disaster strikes (17). These structural conditions have direct public health implications, as energy instability heightens health risks for already vulnerable populations.

At the same time, the case of *Casa Pueblo* underscores the critical role of grassroots resilience in mitigating the health and social impacts of political abandonment. By providing solar energy solutions, healthcare support, and community-driven infrastructure, *Casa Pueblo* actively counters the normalization of crisis governance. Their work demonstrates that community-driven energy initiatives can serve as a public health intervention, protecting medically vulnerable populations in disaster-prone regions. *Casa Pueblo* self-management model shows their commitment to transparency and knowledge-sharing, in contrast to the government’s lack of openness, which limits opportunities for learning from its processes. The significance of *Casa Pueblo’s* effort extends beyond the immediate impact of solar energy adoption. It represents a broader aspiration to strengthen the social fabric of the community, which remains essential for reducing vulnerabilities and managing disasters.

## Data Availability

The raw data supporting the conclusions of this article will be made available by the authors, without undue reservation.
